# Supplementary material for: Transcriptome Analysis Reveals That C17 Mycosubtilin Antagonizes Verticillium dahliae by Interfering with Multiple Functional Pathways of Fungi
Source: Biology (Basel). 2023 Mar 29;12(4):513. doi: 10.3390/biology12040513 (PMC10136297; doi:10.3390/biology12040513)
Supplement: Supplementary file 1 [file biology-12-00513-s001.zip › Supplementary information.pdf]

Supplementary information

Table S1. 2–h and 6–h common C17 mycosubtilin response DEGs. Sheet 1. Genes related to membrane components. Sheet 2. Genes related to DNA replication. Sheet 3. Genes related to the cell cycle. Sheet 4. Genes related to hydrolase activity. Sheet 5. For genes related to oxidoreductase activity, the red font represents the gene enriched in the dioxygenase activity term. Sheet 6. Genes related to energy metabolism.

Table S2. 2–h specific C17 mycosubtilin response DEGs. Sheet 1. Genes related to hydrolase activity. Sheet 2. Genes related to energy metabolism.

Table S3. 6–h specific C17 mycosubtilin response DEGs. Sheet 1. Genes related to transmembrane transport. Sheet 2. Genes related to translation. Sheet 3. Genes related to energy metabolism.

Table S4. Primers used for qRT–PCR.

Figure S1. C17 mycosubtilin affects the spore viability and hyphal microscopic morphology of Vd 991. (A) Growth of Vd 991 in Czapek–Dox broth medium containing different concentrations of C17 mycosubtilin. (B) Vd 991 colony area statistics after growing in different concentrations of C17 mycosubtilin for 72 h. In this experiment, the blank control (CK) group was only supplemented with Czapek–Dox Broth medium but without C17 mycosubtilin and spores; the control group was only supplemented with Czapek–Dox Broth medium and spores but without C17 mycosubtilin. The minimum concentration that inhibited the growth of Vd 991 for 72 h was defined as the MIC. The IC50 was defined as the concentration at which the

colony growth area of Vd 991 was half of that of the control at 72 h. Statistical analyses were performed by t test. The data represent the mean ± SD, $n \geq 3$. ns indicates no significant difference, and **** indicates extreme significance at $P < 0.0001$. (C,D) SEM images of Vd 991 hyphae. Vd 991 hyphae was treated with C17 mycosubtilin at IC50 (C) and MIC (D) concentrations at different times. a, untreated Vd 991 hyphae (control); b1-e1, Vd 991 hyphae treated for 6 h, 12 h, 18 h, and 24 h, respectively. The red-framed area is the area displayed in the next row (b2-e2, and b3-e3, respectively).

Figure S2. The effect of C17 mycosubtilin at the MIC concentration on the morphology and structure of different organelles of Vd 991. (A) TEM images of Vd 991 spores. a, d, untreated Vd 991 spores (control); b, e, Vd 991 spores treated with C17 mycosubtilin for 2 h at MIC concentrations; c, f, Vd 991 spores treated with C17 mycosubtilin for 6 h at MIC concentrations. M, mitochondria; N, nucleus; Ld, lipid droplets; CW, cell wall; dCW, damaged cell wall; PM, plasma membrane; dPM, damaged plasma membrane. (B) Mitochondrial aspect ratio chart. The aspect ratio of spore mitochondria treated with the MIC concentration of C17 mycosubtilin for 0 h, 2 h, and 6 h was calculated. At least 30 mitochondria in each group were counted in each experiment, and the experiment was repeated 3 times. Statistical analyses were performed using one-way ANOVA. No less than 30 mitochondria per group were counted, and the values are the mean ± SD. *** indicates extreme significance at $p < 0.001$.

Figure S3. The conidial necrosis of Vd 991 was analysed by flow cytometry after treatment with C17 mycosubtilin. (A) The spore population of Vd 991 after treatment

with C17 mycosubtilin at the MIC concentration at different times by annexin V-FITC/PI double staining. a, untreated Vd 991 spores (control); b–e, Vd 991 spores treated for 2 h, 6 h, 12 h, and 24 h, respectively; f, summary of spore population data. Each dot represents a cell, and the color represents the cell density. (B) Vd 991 spores treated with C17 mycosubtilin at the MIC concentration at different times by PI staining. a, Untreated Vd 991 spores (control); b–e, Vd 991 spores treated for 2 h, 6 h, 12 h, and 24 h, respectively; f, statistical diagram of spore staining rate at different treatment times. Statistical analysis in this figure is based on one-way ANOVA. Values are mean ± SD, where $n \geq 3$; ns means no significant difference, *** means significant difference at $p < 0.001$, and **** means significant difference at $p < 0.0001$.

Figure S4. Assessment of RNAseq sequencing data quality. (A,B) Principal component analysis (PCA) of transcriptomic samples. Dots of the same color represent each biological repetition in the group, and the distance between dots represents the overall expression difference of the samples; PC1, PC2, and PC3 represent different principal component dimensions, and the numbers in parentheses represent the interpretation degree of principal components. The higher the degree of component interpretation, the greater the overall difference of the samples in the dimension of the principal component vector. (C) Heatmap of the correlation coefficient between samples. M0H6 and M0H2 indicated that the spores grew naturally in Czapek–Dox broth medium without C17 mycosubtilin treatment for 6 h and 2 h, respectively; M3H6 and M3H2 indicated that the spores grew in Czapek–Dox broth medium containing C17 mycosubtilin at the IC50 concentration for 6 h and 2 h, respectively.

Figure S5. Effect of C17 mycosubtilin on gene expression in Vd 991 spores presented with gene expression heatmap. (A) DNA replication-related genes (2–h and 6–h common DEGs). (B) Genes related to the mitotic cell cycle process (2–h and 6–h common DEGs). (C) Downregulated genes related to the cell wall and cell membrane (2–h and 6–h common DEGs). (D) Upregulated genes related to the cell wall and cell membrane (2–h and 6–h common DEGs). (E) Genes related to the translation process (6–h specific DEGs). (F) Downregulated genes related to transmembrane transport activity (6–h specific DEGs). (G) Upregulated genes related to transmembrane transport activity (6–h specific DEGs). M0H6 and M0H2 indicated that the spores grew naturally in Czapek–Dox broth medium without C17 mycosubtilin treatment for 6 h and 2 h, respectively; M3H6 and M3H2 indicated that the spores grew in Czapek–Dox broth medium containing C17 mycosubtilin at the IC50 concentration for 6 h and 2 h, respectively.

Figure S6. Effect of C17 mycosubtilin on gene expression in Vd 991 spores presented with gene expression heatmap. (A) Upregulated genes related to hydrolase activity (2–h and 6–h common DEGs). (B) Downregulated genes related to hydrolase activity (2–h and 6–h common DEGs). (C) Downregulated genes related to oxidoreductase activity (2–h and 6–h common DEGs). (D) Upregulated genes related to oxidoreductase activity (2–h and 6–h common DEGs). (E). Upregulated genes related to hydrolase activity (2–h specific DEGs). (F). Downregulated genes related to hydrolase activity (2–h specific DEGs). M0H6 and M0H2 indicated that the spores grew naturally in Czapek–Dox broth medium without C17 mycosubtilin treatment for

6 h and 2 h, respectively; M3H6 and M3H2 indicated that the spores grew in Czapek–Dox broth medium containing C17 mycosubtilin at the IC50 concentration for 6 h and 2 h, respectively.

Figure S1

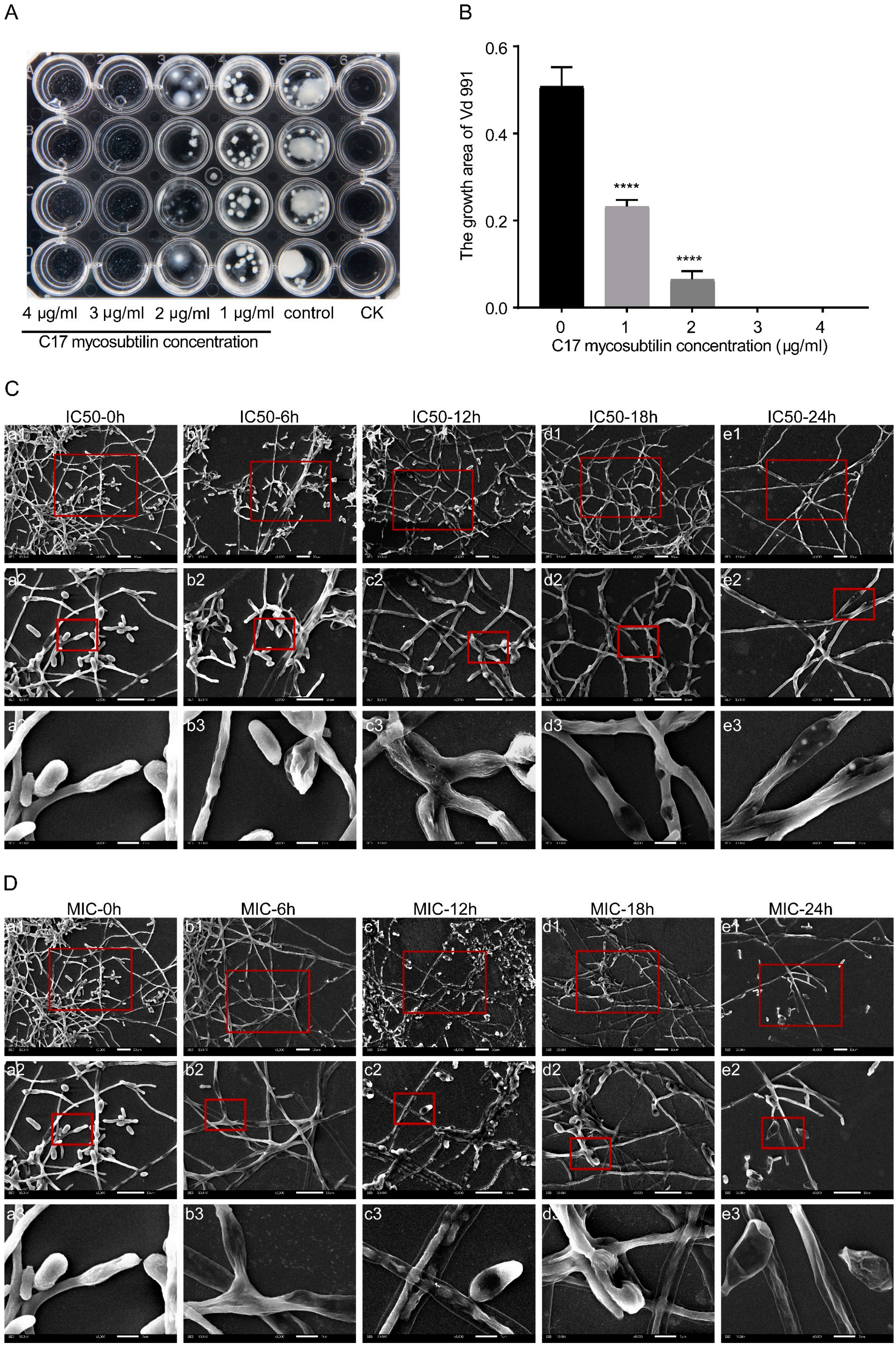
A
4 μg/ml
3 μg/ml
2 μg/ml
1 μg/ml
control
CK
C17 mycosubtilin concentration
B
The growth area of Vd 991
0.6
0.4
0.2
0.0
****
****
0
1
2
3
4
C17 mycosubtilin concentration (μg/ml)
C
IC50-0h
IC50-6h
IC50-12h
IC50-18h
IC50-24h
D
MIC-0h
MIC-6h
MIC-12h
MIC-18h
MIC-24h

Figure S2

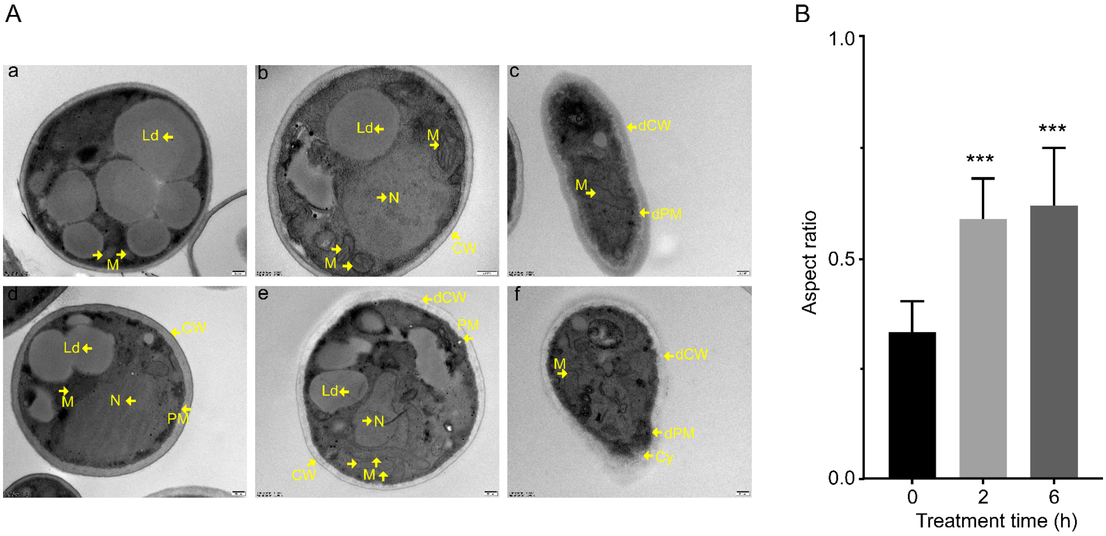

A
a
b
c
d
e
f
Ld
M
N
CW
dCW
dPM
PM
B
1.0
0.5
0.0
Aspect ratio
***
***
0
2
6
Treatment time (h)

Figure S3

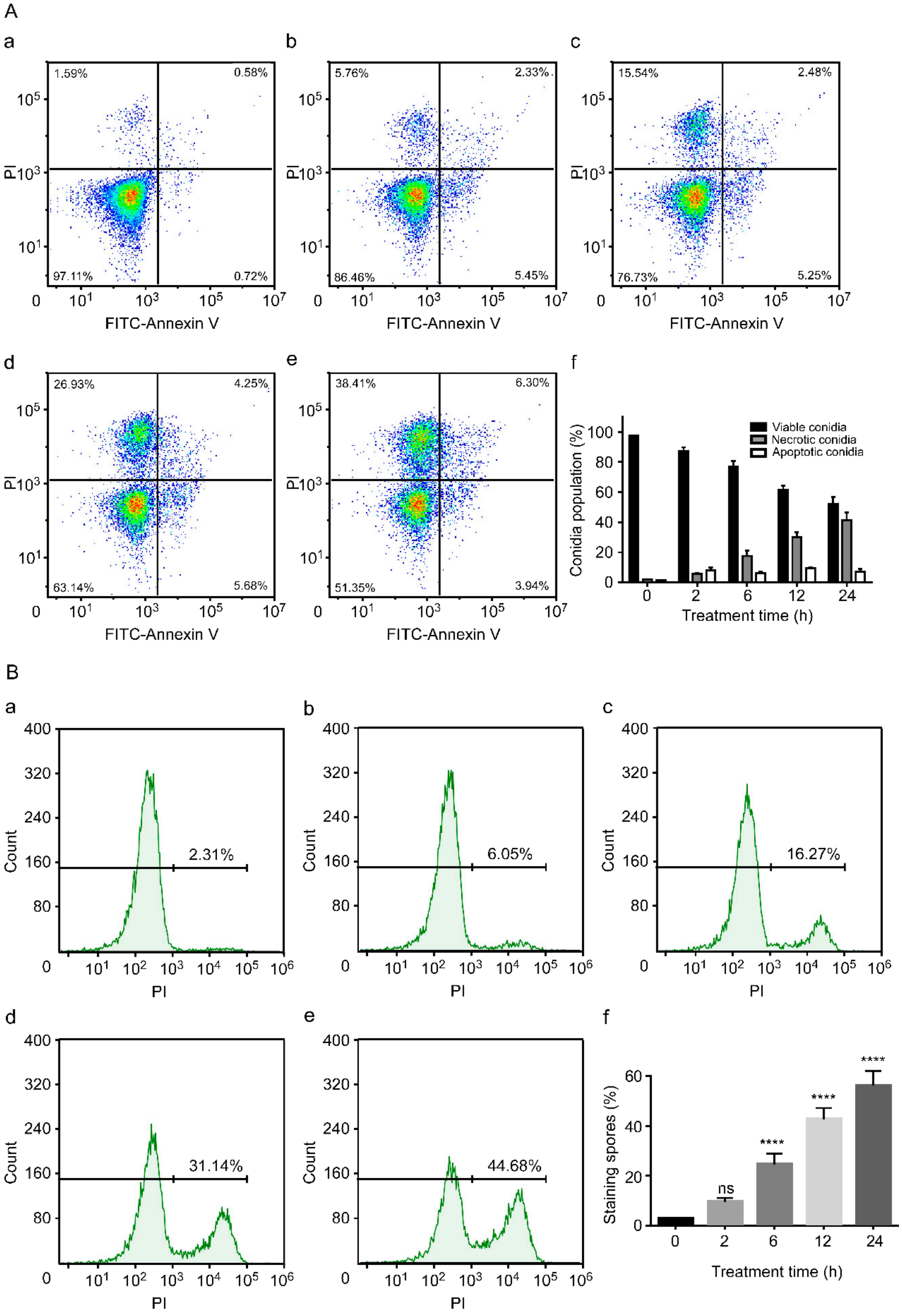

A
a
1.59%
0.58%
97.11%
0.72%
b
5.76%
2.33%
86.46%
5.45%
c
15.54%
2.48%
76.73%
5.25%
d
26.93%
4.25%
63.14%
5.68%
e
38.41%
6.30%
51.35%
3.94%
PI
FITC-Annexin V
f
Viable conidia
Necrotic conidia
Apoptotic conidia
Conidia population (%)
Treatment time (h)
B
a
2.31%
b
6.05%
c
16.27%
d
31.14%
e
44.68%
Count
PI
f
ns
****
Staining spores (%)
Treatment time (h)

Figure S4

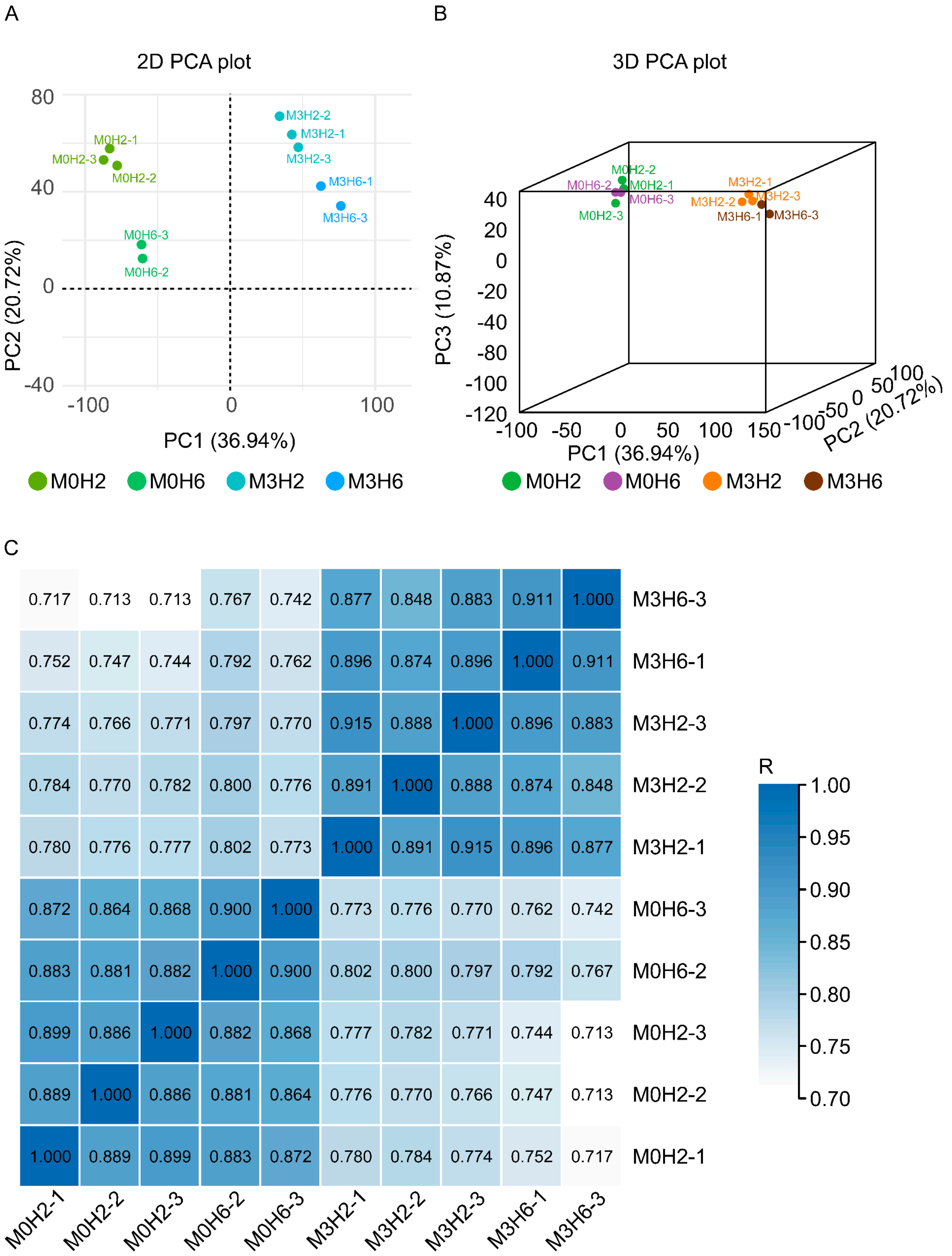
A
2D PCA plot
PC2 (20.72%)
PC1 (36.94%)
80
40
0
-40
-100
0
100
M0H2-1
M0H2-3
M0H2-2
M0H6-3
M0H6-2
M3H2-2
M3H2-1
M3H2-3
M3H6-1
M3H6-3
M0H2 M0H6 M3H2 M3H6
B
3D PCA plot
PC3 (10.87%)
40
20
0
-20
-40
-80
-100
-120
-100 -50 0 50 100 150
PC1 (36.94%)
-100 -50 0 50 100
PC2 (20.72%)
M0H2-2
M0H6-2
M0H2-1
M0H6-3
M0H2-3
M3H2-1
M3H2-3
M3H2-2
M3H6-1
M3H6-3
M0H2 M0H6 M3H2 M3H6
C
0.717 0.713 0.713 0.767 0.742 0.877 0.848 0.883 0.911 1.000 M3H6-3
0.752 0.747 0.744 0.792 0.762 0.896 0.874 0.896 1.000 0.911 M3H6-1
0.774 0.766 0.771 0.797 0.770 0.915 0.888 1.000 0.896 0.883 M3H2-3
0.784 0.770 0.782 0.800 0.776 0.891 1.000 0.888 0.874 0.848 M3H2-2
0.780 0.776 0.777 0.802 0.773 1.000 0.891 0.915 0.896 0.877 M3H2-1
0.872 0.864 0.868 0.900 1.000 0.773 0.776 0.770 0.762 0.742 M0H6-3
0.883 0.881 0.882 1.000 0.900 0.802 0.800 0.797 0.792 0.767 M0H6-2
0.899 0.886 1.000 0.882 0.868 0.777 0.782 0.771 0.744 0.713 M0H2-3
0.889 1.000 0.886 0.881 0.864 0.776 0.770 0.766 0.747 0.713 M0H2-2
1.000 0.889 0.899 0.883 0.872 0.780 0.784 0.774 0.752 0.717 M0H2-1
M0H2-1 M0H2-2 M0H2-3 M0H6-2 M0H6-3 M3H2-1 M3H2-2 M3H2-3 M3H6-1 M3H6-3
R
1.00
0.95
0.90
0.85
0.80
0.75
0.70

Figure S5

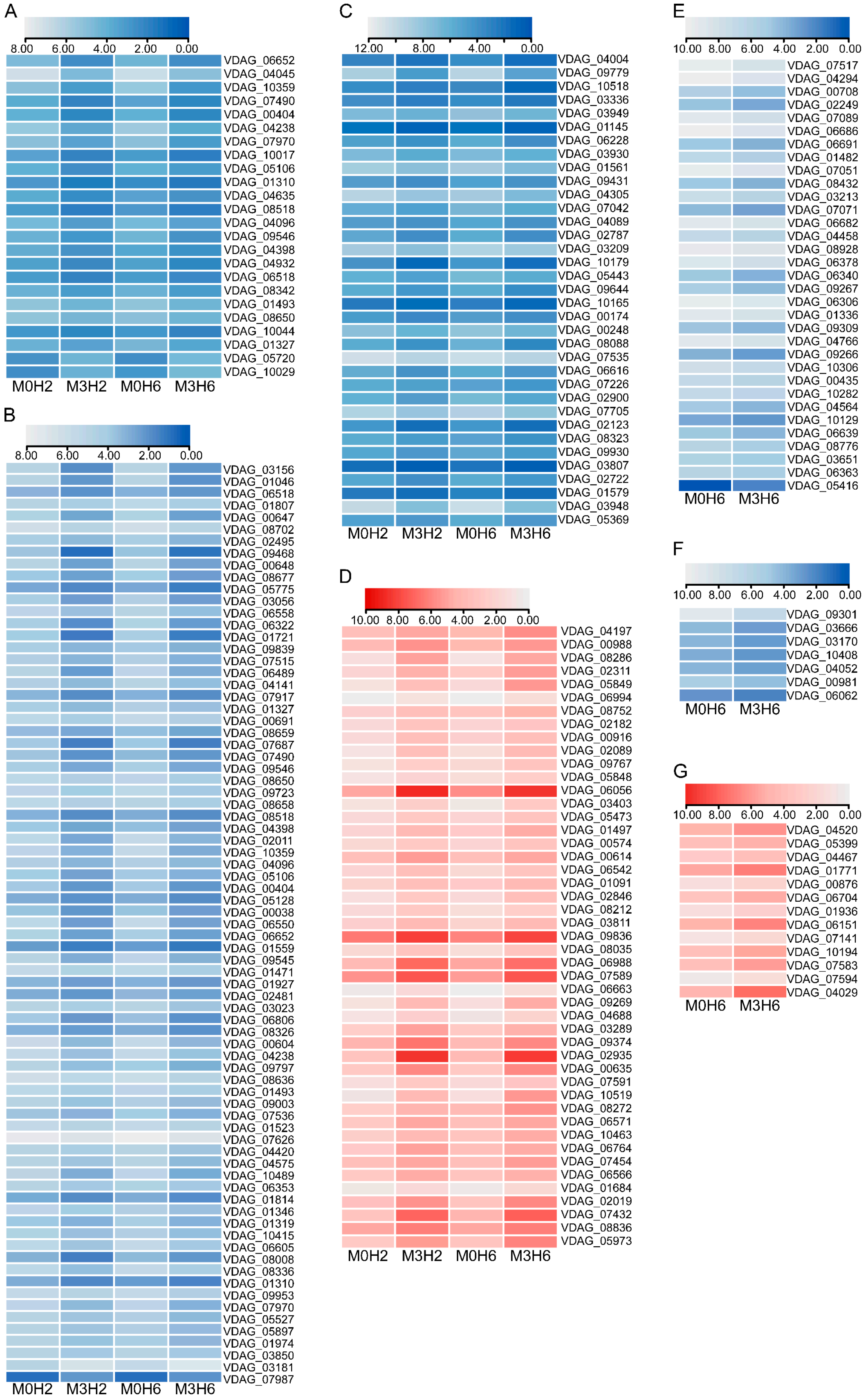
A
8.00 6.00 4.00 2.00 0.00
VDAG_06652
VDAG_04045
VDAG_10359
VDAG_07490
VDAG_00404
VDAG_04238
VDAG_07970
VDAG_10017
VDAG_05106
VDAG_01310
VDAG_04635
VDAG_08518
VDAG_04096
VDAG_09546
VDAG_04398
VDAG_04932
VDAG_06518
VDAG_08342
VDAG_01493
VDAG_08650
VDAG_10044
VDAG_01327
VDAG_05720
VDAG_10029
M0H2 M3H2 M0H6 M3H6
B
8.00 6.00 4.00 2.00 0.00
VDAG_03156
VDAG_01046
VDAG_06518
VDAG_01807
VDAG_00647
VDAG_08702
VDAG_02495
VDAG_09468
VDAG_00648
VDAG_08677
VDAG_05775
VDAG_03056
VDAG_06558
VDAG_06322
VDAG_01721
VDAG_09839
VDAG_07515
VDAG_06489
VDAG_04141
VDAG_07917
VDAG_01327
VDAG_00691
VDAG_08659
VDAG_07687
VDAG_07490
VDAG_09546
VDAG_08650
VDAG_09723
VDAG_08658
VDAG_08518
VDAG_04398
VDAG_02011
VDAG_10359
VDAG_04096
VDAG_05106
VDAG_00404
VDAG_05128
VDAG_00038
VDAG_06550
VDAG_06652
VDAG_01559
VDAG_09545
VDAG_01471
VDAG_01927
VDAG_02481
VDAG_03023
VDAG_06806
VDAG_08326
VDAG_00604
VDAG_04238
VDAG_09797
VDAG_08636
VDAG_01493
VDAG_09003
VDAG_07536
VDAG_01523
VDAG_07626
VDAG_04420
VDAG_04575
VDAG_10489
VDAG_06353
VDAG_01814
VDAG_01346
VDAG_01319
VDAG_10415
VDAG_06605
VDAG_08008
VDAG_08336
VDAG_01310
VDAG_09953
VDAG_07970
VDAG_05527
VDAG_05897
VDAG_01974
VDAG_03850
VDAG_03181
VDAG_07987
M0H2 M3H2 M0H6 M3H6
C
12.00 8.00 4.00 0.00
VDAG_04004
VDAG_09779
VDAG_10518
VDAG_03336
VDAG_03949
VDAG_01145
VDAG_06228
VDAG_03930
VDAG_01561
VDAG_09431
VDAG_04305
VDAG_07042
VDAG_04089
VDAG_02787
VDAG_03209
VDAG_10179
VDAG_05443
VDAG_09644
VDAG_10165
VDAG_00174
VDAG_00248
VDAG_08088
VDAG_07535
VDAG_06616
VDAG_07226
VDAG_02900
VDAG_07705
VDAG_02123
VDAG_08323
VDAG_09930
VDAG_03807
VDAG_02722
VDAG_01579
VDAG_03948
VDAG_05369
M0H2 M3H2 M0H6 M3H6
D
10.00 8.00 6.00 4.00 2.00 0.00
VDAG_04197
VDAG_00988
VDAG_08286
VDAG_02311
VDAG_05849
VDAG_06994
VDAG_08752
VDAG_02182
VDAG_00916
VDAG_02089
VDAG_09767
VDAG_05848
VDAG_06056
VDAG_03403
VDAG_05473
VDAG_01497
VDAG_00574
VDAG_00614
VDAG_06542
VDAG_01091
VDAG_02846
VDAG_08212
VDAG_03811
VDAG_09836
VDAG_08035
VDAG_06988
VDAG_07589
VDAG_06663
VDAG_09269
VDAG_04688
VDAG_03289
VDAG_09374
VDAG_02935
VDAG_00635
VDAG_07591
VDAG_10519
VDAG_08272
VDAG_06571
VDAG_10463
VDAG_06764
VDAG_07454
VDAG_06566
VDAG_01684
VDAG_02019
VDAG_07432
VDAG_08836
VDAG_05973
M0H2 M3H2 M0H6 M3H6
E
10.00 8.00 6.00 4.00 2.00 0.00
VDAG_07517
VDAG_04294
VDAG_00708
VDAG_02249
VDAG_07089
VDAG_06686
VDAG_06691
VDAG_01482
VDAG_07051
VDAG_08432
VDAG_03213
VDAG_07071
VDAG_06682
VDAG_04458
VDAG_08928
VDAG_06378
VDAG_06340
VDAG_09267
VDAG_06306
VDAG_01336
VDAG_09309
VDAG_04766
VDAG_09266
VDAG_10306
VDAG_00435
VDAG_10282
VDAG_04564
VDAG_10129
VDAG_06639
VDAG_08776
VDAG_03651
VDAG_06363
VDAG_05416
M0H6 M3H6
F
10.00 8.00 6.00 4.00 2.00 0.00
VDAG_09301
VDAG_03666
VDAG_03170
VDAG_10408
VDAG_04052
VDAG_00981
VDAG_06062
M0H6 M3H6
G
10.00 8.00 6.00 4.00 2.00 0.00
VDAG_04520
VDAG_05399
VDAG_04467
VDAG_01771
VDAG_00876
VDAG_06704
VDAG_01936
VDAG_06151
VDAG_07141
VDAG_10194
VDAG_07583
VDAG_07594
VDAG_04029
M0H6 M3H6

Figure S6

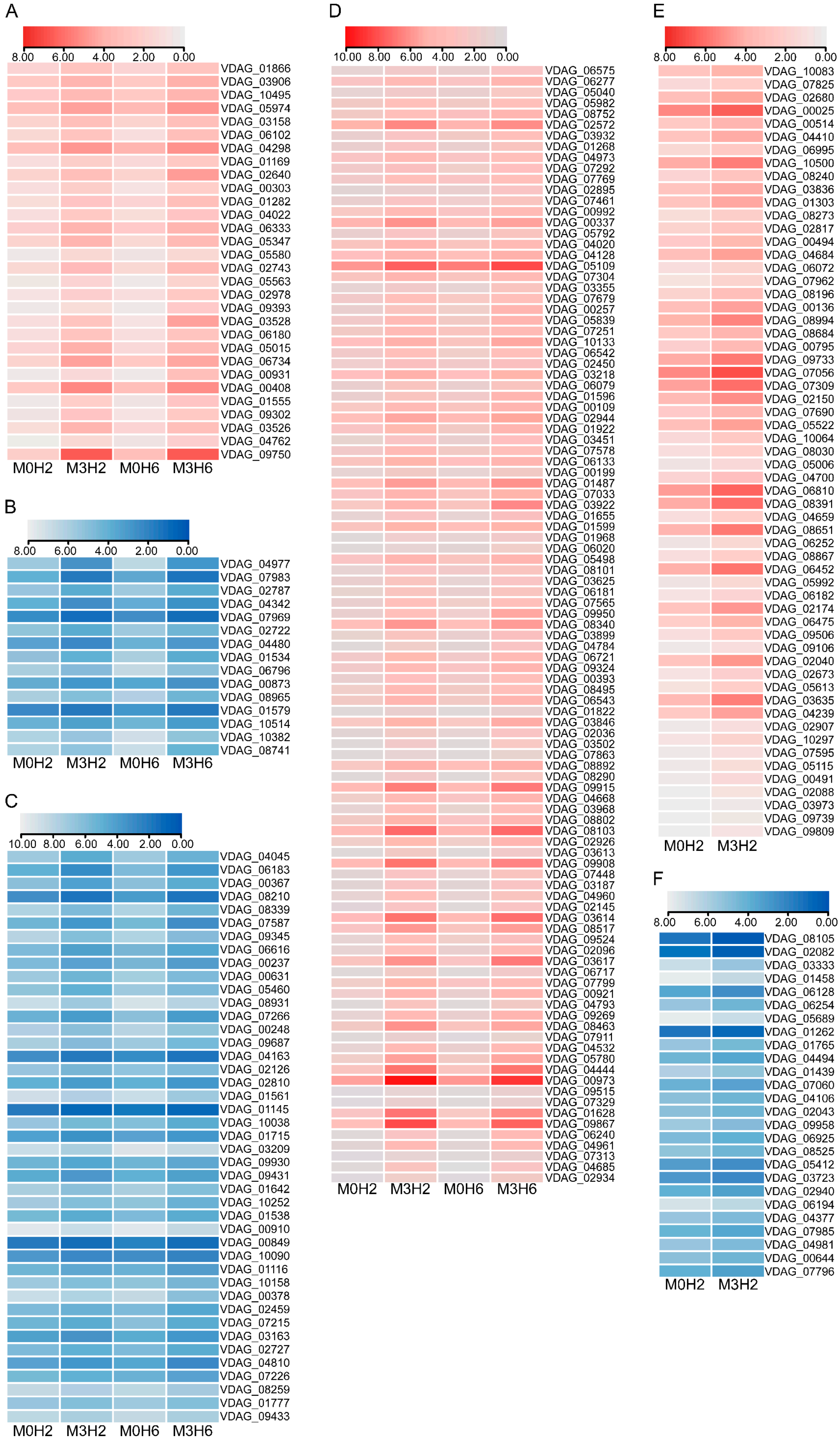
A
8.00 6.00 4.00 2.00 0.00
VDAG_01866
VDAG_03906
VDAG_10495
VDAG_05974
VDAG_03158
VDAG_06102
VDAG_04298
VDAG_01169
VDAG_02640
VDAG_00303
VDAG_01282
VDAG_04022
VDAG_06333
VDAG_05347
VDAG_05580
VDAG_02743
VDAG_05563
VDAG_02978
VDAG_09393
VDAG_03528
VDAG_06180
VDAG_05015
VDAG_06734
VDAG_00931
VDAG_00408
VDAG_01555
VDAG_09302
VDAG_03526
VDAG_04762
VDAG_09750
M0H2 M3H2 M0H6 M3H6
B
8.00 6.00 4.00 2.00 0.00
VDAG_04977
VDAG_07983
VDAG_02787
VDAG_04342
VDAG_07969
VDAG_02722
VDAG_04480
VDAG_01534
VDAG_06796
VDAG_00873
VDAG_08965
VDAG_01579
VDAG_10514
VDAG_10382
VDAG_08741
M0H2 M3H2 M0H6 M3H6
C
10.00 8.00 6.00 4.00 2.00 0.00
VDAG_04045
VDAG_06183
VDAG_00367
VDAG_08210
VDAG_08339
VDAG_07587
VDAG_09345
VDAG_06616
VDAG_00237
VDAG_00631
VDAG_05460
VDAG_08931
VDAG_07266
VDAG_00248
VDAG_09687
VDAG_04163
VDAG_02126
VDAG_02810
VDAG_01561
VDAG_01145
VDAG_10038
VDAG_01715
VDAG_03209
VDAG_09930
VDAG_09431
VDAG_01642
VDAG_10252
VDAG_01538
VDAG_00910
VDAG_00849
VDAG_10090
VDAG_01116
VDAG_10158
VDAG_00378
VDAG_02459
VDAG_07215
VDAG_03163
VDAG_02727
VDAG_04810
VDAG_07226
VDAG_08259
VDAG_01777
VDAG_09433
M0H2 M3H2 M0H6 M3H6
D
10.00 8.00 6.00 4.00 2.00 0.00
VDAG_06575
VDAG_06277
VDAG_05040
VDAG_05982
VDAG_08752
VDAG_02572
VDAG_03932
VDAG_01268
VDAG_04973
VDAG_07292
VDAG_07769
VDAG_02895
VDAG_07461
VDAG_00992
VDAG_00337
VDAG_05792
VDAG_04020
VDAG_04128
VDAG_05109
VDAG_07304
VDAG_03355
VDAG_07679
VDAG_00257
VDAG_05839
VDAG_07251
VDAG_10133
VDAG_06542
VDAG_02450
VDAG_03218
VDAG_06079
VDAG_01596
VDAG_00109
VDAG_02944
VDAG_01922
VDAG_03451
VDAG_07578
VDAG_06133
VDAG_00199
VDAG_01487
VDAG_07033
VDAG_03922
VDAG_01655
VDAG_01599
VDAG_01968
VDAG_06020
VDAG_05498
VDAG_08101
VDAG_03625
VDAG_06181
VDAG_07565
VDAG_09950
VDAG_08340
VDAG_03899
VDAG_04784
VDAG_06721
VDAG_09324
VDAG_00393
VDAG_08495
VDAG_06543
VDAG_01822
VDAG_03846
VDAG_02036
VDAG_03502
VDAG_07863
VDAG_08892
VDAG_08290
VDAG_09915
VDAG_04668
VDAG_03968
VDAG_08802
VDAG_08103
VDAG_02926
VDAG_03613
VDAG_09908
VDAG_07448
VDAG_03187
VDAG_04960
VDAG_02145
VDAG_03614
VDAG_08517
VDAG_09524
VDAG_02096
VDAG_03617
VDAG_06717
VDAG_07799
VDAG_00921
VDAG_04793
VDAG_09269
VDAG_08463
VDAG_07911
VDAG_04532
VDAG_05780
VDAG_04444
VDAG_00973
VDAG_09515
VDAG_07329
VDAG_01628
VDAG_09867
VDAG_06240
VDAG_04961
VDAG_07313
VDAG_04685
VDAG_02934
M0H2 M3H2 M0H6 M3H6
E
8.00 6.00 4.00 2.00 0.00
VDAG_10083
VDAG_07825
VDAG_02680
VDAG_00025
VDAG_00514
VDAG_04410
VDAG_06995
VDAG_10500
VDAG_08240
VDAG_03836
VDAG_01303
VDAG_08273
VDAG_02817
VDAG_00494
VDAG_04684
VDAG_06072
VDAG_07962
VDAG_08196
VDAG_00136
VDAG_08994
VDAG_08684
VDAG_00795
VDAG_09733
VDAG_07056
VDAG_07309
VDAG_02150
VDAG_07690
VDAG_05522
VDAG_10064
VDAG_08030
VDAG_05006
VDAG_04700
VDAG_06810
VDAG_08391
VDAG_04659
VDAG_08651
VDAG_06252
VDAG_08867
VDAG_06452
VDAG_05992
VDAG_06182
VDAG_02174
VDAG_06475
VDAG_09506
VDAG_09106
VDAG_02040
VDAG_02673
VDAG_05613
VDAG_03635
VDAG_04239
VDAG_02907
VDAG_10297
VDAG_07595
VDAG_05115
VDAG_00491
VDAG_02088
VDAG_03973
VDAG_09739
VDAG_09809
M0H2 M3H2
F
8.00 6.00 4.00 2.00 0.00
VDAG_08105
VDAG_02082
VDAG_03333
VDAG_01458
VDAG_06128
VDAG_06254
VDAG_05689
VDAG_01262
VDAG_01765
VDAG_04494
VDAG_01439
VDAG_07060
VDAG_04106
VDAG_02043
VDAG_09958
VDAG_06925
VDAG_08525
VDAG_05412
VDAG_03723
VDAG_02940
VDAG_06194
VDAG_04377
VDAG_07985
VDAG_04981
VDAG_00644
VDAG_07796
M0H2 M3H2
