# Supplementary material for: Transcriptome Analysis Reveals That C17 Mycosubtilin Antagonizes Verticillium dahliae by Interfering with Multiple Functional Pathways of Fungi
Source: Biology (Basel). 2023 Mar 29;12(4):513. doi: 10.3390/biology12040513 (PMC10136297; doi:10.3390/biology12040513)
Supplement: Supplementary file 1 [file biology-12-00513-s001.zip › Table S4.pdf]

Table S4 Primers used for qRT–PCR

NO.	Primer names	Sequences (5'to3')
1	03948L	TTCTCATCGTCTGGGTTTCTG
2	03948R	GAGAATAGCGAGGGTGAAGC
3	09779L	CAAGTCCGTGAAGACCATCTC
4	09779R	GAGGGAGCAGAGGGAGTAG
5	03930L	GTTCTCCACTACCTGCCATTC
6	03930R	TGGCGTACTGGATTGTCTTG
7	06452L	GGCGACAACTTTTACAAGCAG
8	06452R	GAGTGGTCGTAGTCCTTTTGG
9	05522L	ATGCCTCCGAGTTTATGACC
10	05522R	GGGCTATCGTACTTTTCCTGG
11	07309L	TTTGGTGTTCTCGTCATCCC
12	07309R	TTTGATAGCCTGGAAGAGCG
13	07051L	GTTGGTATCGTGGGCTACATC
14	07051R	GTACCAGTTCCTGTAGAAGCG
15	08928L	AGATCCACGAGTACCTCTTCC
16	08928R	CGACTGGAGAGCCTTGATG
17	07089L	GTCCTGAAGTTCGCCCAC
18	07089R	GGGTGATGTAGTTGGTGAAGG
19	β-tubulin L	AGTTCACTGCCATGTTCCG
20	β-tubulin R	TCATGTTGAACTCAGCCTCAG
